# Novel Video Surveillance-Based Fire and Smoke Classification Using Attentional Feature Map in Capsule Networks

**DOI:** 10.3390/s22010098

**Published:** 2021-12-24

**Authors:** Muksimova Shakhnoza, Umirzakova Sabina, Mardieva Sevara, Young-Im Cho

**Affiliations:** 1 Department of IT Convergence Engineering, Gachon University, Sujeong-gu, Seongnam-si 461-701, Gyeonggi-do, Korea; shakhnoza02@gachon.ac.kr (M.S.); sabina@gachon.ac.kr (U.S.); 2 Department Information Security, Tashkent University of Information Technologies Named after Muhammad al-Khwarizmi Tashkent, Tashkent 100200, Uzbekistan; sevara@tuit.uz; 3 Department of Computer Engineering, Gachon University, Sujeong-gu, Seongnam-si 461-701, Gyeonggi-do, Korea

**Keywords:** capsule network, attention feature map, smoke detection, fire detection, deep learning, artificial intelligence, classification

## Abstract

A fire is an extraordinary event that can damage property and have a notable effect on people’s lives. However, the early detection of smoke and fire has been identified as a challenge in many recent studies. Therefore, different solutions have been proposed to approach the timely detection of fire events and avoid human casualties. As a solution, we used an affordable visual detection system. This method is possibly effective because early fire detection is recognized. In most developed countries, CCTV surveillance systems are installed in almost every public location to take periodic images of a specific area. Notwithstanding, cameras are used under different types of ambient light, and they experience occlusions, distortions of view, and changes in the resulting images from different camera angles and the different seasons of the year, all of which affect the accuracy of currently established models. To address these problems, we developed an approach based on an attention feature map used in a capsule network designed to classify fire and smoke locations at different distances outdoors, given only an image of a single fire and smoke as input. The proposed model was designed to solve two main limitations of the base capsule network input and the analysis of large-sized images, as well as to compensate the absence of a deep network using an attention-based approach to improve the classification of the fire and smoke results. In term of practicality, our method is comparable with prior strategies based on machine learning and deep learning methods. We trained and tested the proposed model using our datasets collected from different sources. As the results indicate, a high classification accuracy in comparison with other modern architectures was achieved. Further, the results indicate that the proposed approach is robust and stable for the classification of images from outdoor CCTV cameras with different viewpoints given the presence of smoke and fire.

## 1. Introduction

Early fire detection is considered a challenging yet important task, considering its direct impact on human safety and the environment. State-of-the-art technology requires appropriate solutions for detecting fires during its earliest possible stage to avoid the possibility of harming human beings [1].

Fire control has always been a challenge to countries around the world. Fires can become uncontrollable, particularly in developing countries, owing to a lack of financial resources required to predict and control the likelihood of such events. Preventing fire events is considered to be of the highest priority owing to unrecoverable damage to populations and even an entire country. Conventionally, fires can be detected using sensory systems that define changes in the presence of smoke or temperature within a compartment. However, not all modern surveillance systems can cover a vast area and provide detailed information regarding the location or level of a fire.

According to the U.S. Fire Administration (USFA), 1.4 million fire incidents were reported by the National Fire Protection Association in the US in 2020. As a result of such fires, 3500 deaths, 15,200 injuries, and approximately USD 21.9 billion in damage occurred [2].

In addition, thousands of closed-circuit television (CCTV) systems have been installed in public locations by national and local authorities. In 2016, 74,000 cameras were installed in the geographically vital areas of South Korea; however, by 2020, this number had increased substantially to 1.34 million. The main aim of installing such cameras is to ensure the safety of the public from unwanted events such as accidents and fires. The ubiquitous use of CCTV systems has helped decrease crime by 45% in areas where such systems have been installed [3].

Deep learning is a popular method for processing the massive number of CCTV images and creating models for identifying unwanted events such as fires and theft. CCTV technologies coupled with deep learning algorithms can help control crime significantly, monitoring specific zones such as kindergartens and traffic areas [4].

Modern CCTV cameras and surveillance systems can use deep learning and similar technologies to detect the presence of fires and smoke at their earliest stages. The ability of CCTV cameras to monitor different types of catastrophes has been proven in numerous studies [5,6,7]. An intelligent CCTV system can eliminate disasters such as smoke and fire or detect break-ins and other abnormal events. Thus, CCTV is an effective tool for smart cities and societies, contributing to a safe and healthy environment.

Nonetheless, the frequent utilization of CCTV within a range of monitoring areas cannot guarantee fire detection during the early stages. Thus, the threat to human life remains in such cases. In other words, fire and smoke detection systems are expensive and inconvenient in terms of indoor installation, and have proven to be less accurate when the image viewpoint changes. Although deep learning algorithms generate results with maximum accuracy in different applications, such as object detection and face recognition, the real-time prediction of fire events remains in a preliminary state and is therefore worth investigating to achieve a state-of-the-art capability.

Fire detection and notification can be achieved through two basic approaches, i.e., sensor- and vision-based methods. A sensor-based approach requires sophisticated equipment such as infrared, smoke, and temperature sensors. As a downside of this approach, it is expensive with outrageously high installation costs. Moreover, such sensors are low-powered devices and thus induce a time delay and misleading alarms.

By contrast, vision-based methods are territorial and the cost is lower than that of sensor-based approaches. The primary goal is to use regular RGB images or videos and to deliver more detailed information in fire detection. In addition, CCTV surveillance systems are already installed in most public locations, which can help in reducing the installation cost. Moreover, vision-based cameras can detect fires much faster owing to high-performing computing abilities and GPUs. Finally, vision-based cameras can observe larger areas that help diagnose fires as early as possible to prevent flames.

The two approaches above coupled with state-of-the-art deep learning algorithms can be used to build an effective model to predict a fire with high accuracy and a faster response time. There are a variety of massively popular deep learning algorithms used in predicting patterns and features in a given image. For example, convolutional neural networks (CNNs) and newer algorithms based on a CNN are popularly used for predicting diseases, through irregular patterns in medical imaging. In this paper, we used the capsule network baseline (CNB) algorithm, which is a variant of the base CNN.

The CNB architecture includes a first digit capsule layer, second convolutional layer, and third primary capsule layer [8]. This approach uses small groups of neurons called capsules. Each of these capsules is designed to detect a specific feature in the image and recognize it in different scenarios, for example, at different angles. The capsules, in turn, form layers to identify objects in video or images. When several capsules in one layer make the same decision, they activate another capsule at a higher layer. This process continues until the network can infer what it is seeing.

**Figure 1:**
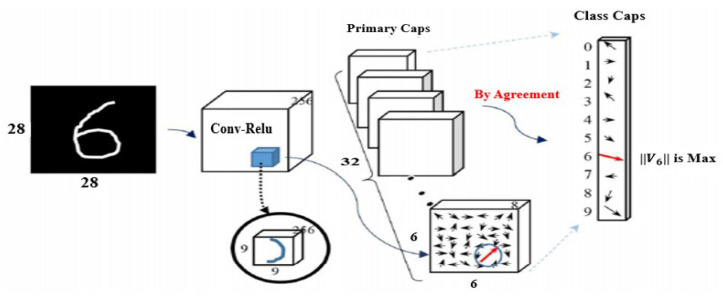
Capsule network architecture.

As shown in Figure 1, the CNB architecture includes a feature extraction layer using convolutions and a primary module composed of several capsule layers followed by a classification (digit) layer. The information held in the primary capsule block is given to the classification (digit) layer, which uses a dynamic routing method stated above, and this method is called routing by agreement. Furthermore, the coupling coefficients among the capsules in the primary and classification (digit) capsule layers are renovated to increase the classification performance. The output of the classification capsule layer is a class capsule of classes, and the norm of every class indicates the foretelling capability for every class.

Our proposed model can be used to represent an essential role in advance. Primary classification in this domain requires positional relations among features for achieving an optimal performance.

Using our architecture, we can analyze the spatial relationships among the features and relevant locations in an input by using a capsule network structure. 

We propose the application of two broad technical modifications to the original dynamic routing algorithm under the following conditions:

1. A concise overview of the attention feature map is given. We use an attention feature map based on the capsule network to build our architecture. As we use an attention feature map, we develop a robust capsule network-based approach that takes a lower layer and routes to a higher layer within a limited spatially local window.
2. We use large sizes to learn the features from fire input images. The introduction of new capsule-type transformation matrices uses portions of the grid. These changes support our input on large image sizes with convolutional capsules having a pixel resolution of up to 512 × 512. Details are provided in Section 3.
3. We propose an attention feature map for modeling multi-level reliability at large distances between image regions by combining low- and high-level capsule features. We vindicated this in our experiments described in Section 5.

The paper is structured as follows: The review of some related studies is presented in Section 2. Section 3 presents a detailed description of the classification of our methods and materials. The specifics of the dataset used in our experiments are detailed in Section 4. Our experiments and the results achieved are given in Section 5. Subsequently, the conclusions and future directions are presented in Section 6.

## 2. Related Work

In this paper, we discuss the research conducted in the field of fire and smoke detection, divided into two main approaches, i.e., computer vision and deep learning.

### 2.1. Computer Vision Based on Smoke and Fire

Detecting fine smoke or fire at a farther distance in an uncertain CCTV environment in the early stage is vital for timely intervention to avoid large-scale damage. Various tools and methods have been used to recognize smoke and fires based on image processing algorithms. Most smoke and fire detection methods use sensors, such as outdoor CCTV systems, which are estimated to detect the presence of smoke or flames. The main limitations of sensors are their minimal range, and an outdoor environment requires widespread detection systems to cover all areas. As a result, they can only identify fires or flames near a designated location. Initially, many researchers endeavored to develop handcrafted techniques for fire detection by concentrating on the action and color properties of flame detection.

Although a wide number of studies have been carried out, many focused on the localization level and hazardous fire and smoke. Recognizing fire at an early stage is a significantly important matter. State-of-the-art technologies require relevant fire detecting systems that can help prevent the occurrence of numerous fire accidents worldwide.

Primarily, experts have focused on the motion and color features of fire detection to build customized algorithms for fire alarm systems. In [9], a method based on Markov002 was applied. The study in [10] developed a camera technique for fire movements and static residential fire detection, which uses the color, boundary, length, and overall shape of the fire. A tiny flame, such as a candle, is used as an afterthought in this approach. Such an approach can have a significant difficulty in early flame detection because it removes and then applies fire development features for an assessment. In this study, the method combination HSV and YCbCr [11] was proposed. This technique requires a further transformation of the color area and is preferable to utilizing a single-color area technique. However, the authors solely employed the static features of the fire. The technique is unstable and fragile. Although another method [12] used hydrogen sensors to improve traditional fire detection systems and to increase the accuracy of fire detection, they shortened the range of sensitivity. Moreover, to detect moving pixels in an image, the authors in [13] proposed a method for estimating the background of a Gaussian mixture. This method defines fire areas based on their color patterns, and then conducts a wavelet analysis in both the space and time domains. Thus, it can analyze the capacity of high-frequency activities in an area. Similar to the previous method, this detection system also has computing problems limiting its practical usage. An efficient method of fire detection proposed in [14] improves a traditional fire detection method using flickering algorithms installed into the scheme to indicate the flame in color video sequences incrementally. In [15], the authors tried to improve the Gaussian model by using motion-based and multicolor detection and obtained good experimental results. Despite this, the method could not be applied to everyday life, because of the high computational time required. As a result, the test results indicated that the proposed algorithms are practical, solid, and efficient. 

In [16,17], the authors proposed a fire detection model with higher accuracy. Their method operates with different types of flame characteristics. An approach following the fire pixel detection technique based on ICA K-medoids, considered the foundation for practical use, has also been presented [18]. In [19], a new color-differentiated conversion matrix system resistant to false positives was demonstrated. Another group of researchers [20] introduced a new low-cost camera with beneficial smoke and flame detecting features for RGB and HSV. However, this camera for use went through limitations in-camera for popularity and application. In [21], owing to the limitations of RGB cameras, the authors used an ultra-spectral camera to control factors that cannot discern between common light (halogen or LED) and a flame. Although the results are promising, there may be certain limitations, such as the higher cost of the camera. The researchers proposed the use of a flame flicker and color sign for fire detection [22]. In addition, in [23], a method based on the radiation domain was introduced from a combination of feature models.

Nonetheless, these methods require the domain knowledge of smoke and fire in the images, which is crucial for exploring hand-crafted features, and they cannot reflect the spatially and temporally involved aspects in smoke and fire outdoors. Moreover, most conventional methods only use a still image or consecutive pairs of frames to detect a fire. Hence, they reflect the short-term dynamic action of the fire rather than the longer-term dynamic action.

### 2.2. Deep Learning-Based Vision for Smoke and Fire Detection

All of the research studies on smoke and fire detection mentioned above differ from those based on computer vision in many different ways.

To begin with, deep learning algorithms were used to conduct an automatic feature extraction from a tremendous quantity of data for training, as well as discriminative characteristics studied using a neural network for fire and smoke detection. From another perspective, deep neural networks can easily be introduced into many other spheres of life. Finally, they can be widely used in constructing a robust dataset and achieving an efficient network structure.

From the discussions above, although video-based fire detection has been studied and has rapidly matured with multidisciplinary technology used to solve the existing limitations of the modern method, several problems remain. In comparison to the image type used in an experiment, a camera image cannot always have rich color information, which can result in a higher rate of false negatives. The false positive indicator can increase, while the algorithm involves fewer fire attributes. Therefore, traditional fire detection must be optimized given the practicality. By contrast, the DL-based approach has the advantage of an automatic extraction of the characteristics. The process is much more efficient and reliable than conventional image processing technologies.

However, this deep learning approach requires many heavy calculations during training and applies hardware to conduct specific tasks and training. For fire detection, distributing the algorithm function on heavy equipment, such as personal computers, is useless because the unit must be similar in terms of existing fire detectors and cost. Various deep learning approaches for fire detection have been proposed [24] through studies on forest fire alarms conducted using fire patches with a fine-tuned pre-released CNN, called “Alexnet” [25,26]. In [27], CNN-based fire detection approaches VGG-16 [28], ResNet-50 [29], and Yolo3 [30] were proposed as a reference architecture.

CCTV and video alert systems can help decrease the detection time compared to other available sensors in interior and exterior scenarios. Surveillance cameras can monitor the amplification without any transport slowdowns that traditional “point” sensors suffer from. CCTV cameras are mainly suitable for observing fire in passenger cars or homes, offices, and factories within a 100 m distance. In the case of forest or rural areas, other more advanced technologies must be used and optimized for scenes observing distances of several kilometers. Numerous studies on the detection of fire in videos [31,32,33] have been recently suggested in the field of image/video classification.

CapsNet has been recommended as a powerful functional extraction technology and robust model structure. As a result, traditional computer vision methods are being replaced by deep learning methods. Our proposed method adopts models that classify smoke and fire in an image/video. An incorrect categorization of images or videos increases the incorrect fire alarm rate owing to changes in the perspective deformations, shadow, and brightness. We detected a template that uses fire and smoke, showing images based on an attentional feature map using CapsNet to learn and extract the powerful attributes of the frame.

## 3. Materials and Methods

Proposed Network Architecture

**Figure 2:**
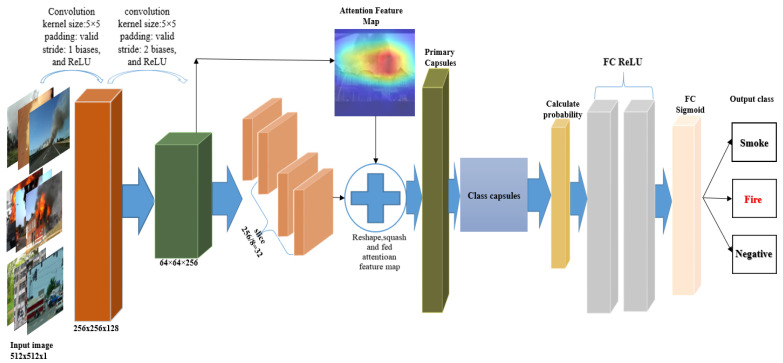
Architecture of the proposed classification model.

As demonstrated in Figure 2, the input size from the capsular network has a pixel resolution of 512 × 512 when passing through a convolutional layer, creating k × k feature maps of the exact spatial dimensions. This first set of capsule outputs form a k × k vector, where we have one capsule type with a 512 × 512 mesh of capsules. This is followed by the first layer of the convolutional capsule. In the next step, we generalize our convolutional capsules and routing to any layer in the network. In the layer, there are many types of capsules.

(1) $$C^{l}=\left\{C_{1}^{l},C_{2}^{l},\ldots ,C_{n}^{l}|n\epsilon N\right\}$$

For each $c_{i}^{\iota }$ $\epsilon \;T^{\iota }$, type of capsule, there is a height, weight, and grid $z^{l}\;$size of low-level capsules,

(2) $$L=\left\{l_{1,1},\ldots ,l_{1,w^{l}},\ldots ,c_{h^{l},1},\ldots ,c_{h^{l},w^{l}}\right\}$$

The height and weight are the spatial dimensions of the output layer i−1, and there are i+1 capsule types at the next level of the network.

(3) $$C^{l+1}=\left\{c_{1}^{l+1},\;c_{2}^{l+1},\ldots ,c_{m}^{l+1}|m\epsilon Ν\right\}$$

where each layer $c_{i}^{l+1}\;\epsilon \;C^{l+1}\;$of the network capsule has the weight, altitude, and $z^{l+1}$ size of the high-level capsules

(4) $$H=\left\{h_{1,1\ldots ,}h_{1,\;w^{l+1},1\ldots ,}h_{h^{l+1,}1,\ldots ,}h_{h^{l+1},\;w^{l+1}\;\;}\right\}$$

In each capsule $c_{i}^{l+1}\;\epsilon \;C^{l+1}\;$of the convolution, the high-level part $h_{x,y}\;\epsilon \;H$ receives sets of prediction vectors:

(5) $$\left\{\hat{v}{\;}_{x,y|c_{1}^{l}},\hat{v}{\;}_{x,y|c_{2}^{l}},\ldots ,\;\hat{v}{\;}_{x,y|c_{n}^{l}}\right\}$$

This set of vectors is defined as multiplying the matrix between the studied conversion matrix $M{\;}_{c_{j}^{l+1}}$ for this type of high-level capsule and the subnet of low-level capsule output data $V_{x,y|c_{i}^{l}}$. Equation (5) is within the user-defined core with the center at position (x, y) in the layer, and therefore:

(6) $$\hat{v}{\;}_{x,y|c_{i}^{l}=}M_{c_{j}^{l+1}}\times V_{x,y|c_{i}^{l}},\;\;\;\;\;\;\;\;\;\forall \;\;c_{i}^{l}\;\epsilon \;C^{l}$$

Each $V_{x,y|c_{i}^{l}}$ has the form *j*, where $k_{h}\times k_{w}\times z^{l}$ is the size of the user-defined kernel for capsules for all types. Each matrix $M{\;}_{c_{j}^{l+1}}$ is shaped with $k_{h}\times k_{w}\times z^{l}\times z^{l+1}$. Thus, in Equation (6), each $\hat{v}{\;}_{x,y|c_{1}^{l}}$ is the dimension vector $z^{l+1}$, which will be used to form high-level capsules.

The same conversion matrix is used in all spatial areas within this capsule type to drastically reduce the number of parameters to be studied. The values of these transformation matrices for each capsule in the layer are studied using the reverse propagation algorithm with a controlled loss function.

**Figure 3:**
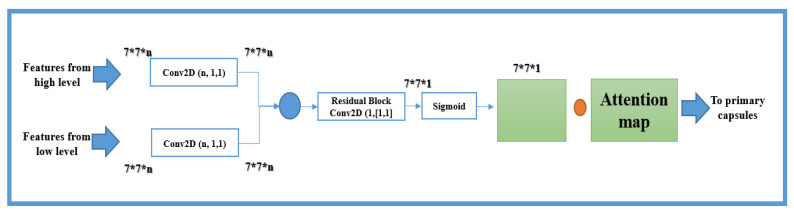
Attention feature map.

The output feature matrix is extracted from the CapsNet high and low convolutional layer and then passed to the attention feature map (Figure 3). The objects at all levels are then combined and transferred to the residual network for achieving a convolution and a standard map of objects.

(7) $$F_{map}=\{Conv\left(\left[F_{L},F_{H}\right]\right)$$

Here, the mask layers of a low-level capsule layer and the high-level elements from the layers of a high-level capsule are used. This helps the capsule network model connect remote regions and balance between efficiency and long-term dependencies by providing a weighted sum of features across all locations in the image. We determine a non-local operation as:

(8) $$\eta_{ij}\left(x\right)=f{\left(x_{i}\right)}^{T}m\left(x_{j}\right),\;f\left(x_{i}\right)=W^{g},\;\;\;m\left(x_{j}\right)=F_{map}.$$

where $W_{g}C^{rxr}$ and$\;F_{map}\epsilon C^{rxr}$ are the learned weight matrices, as illustrated in Figure 3; high- and low-level capsule networks feature a map output inserted into the convolutional layers with a kernel size of *5* × *5*, followed by ReLU, and learns a unique weight tensor for attention feature maps. In particular, the learning of unique weight tensors is formulated as:

(9) $$M=\sigma \left(W^{g}\times F_{map}+b\right)$$

where *M* denotes the weight tensor corresponding to the input map.

After the second convolution, the sigmoid function processes the resulting weight tensor, identifies the protruding areas, and removes the function responses to preserve the activation units. The original feature maps are then combined with each weight tensor by performing an element-wise multiplication operation that results in a weighted feature map. Finally, the element-wise addition operation combines the weighted feature maps to create the final weighted summary feature map. The final output attention feature map is formulated as follows:

(10) $$A=M\times F_{map}$$

The final result of attention map *A* is then transferred to the entry-level. The output vector of capsule *j* will be *vj*. The vector length, which means the probability that a particular object is at a given location in the image, must be between 1 and zero. To ensure this, we used a compressing function that stores information about the location of the object. Short vectors are reduced to zero, and long vectors are reduced to less than 1. The abbreviation function is described as follows:

(11) $$\upsilon_{j}=\frac{||\Sigma_{i}t_{ij}W_{ijA}||{}^{2}}{\left(1+||\Sigma_{i}t_{ij}W_{ijA}||{}^{2}\right)}\frac{\Sigma_{i}t_{ij}W_{ijA}}{||\Sigma_{i}c_{ij}W_{ijA}||}$$

All capsules are in the layer above *j* and capsule *i*, where *W_ij_* is the weight matrix, and *t_ij_* is the coupling coefficients between them, as shown in Equation (11), and is considered through iterative dynamic routing steps:

(12) $$t_{ij}=\frac{\exp\left(b_{ij}\right)}{\Sigma_{i}\exp\left(b_{ij}\right)}$$

Here, $b_{ij}\;$is the logarithm of the prior probabilities that capsule $i^{th}$ should be connected to capsule $j^{th}$. In addition, the $v_{j}$ vector is used to obtain the reconstructed image during training, which provides the highest coupling factor. Here, $t_{ij}$ runs the correct $v_{j}$ through two fully connected ReLU layers. The loss of restoration of the $L_{R}\left(I,\hat{I}\right)$ architecture is defined as follows:

(13) $$L_{R}\left(I,\hat{I}\right)=||I-{\hat{I}||}_{2}^{2}$$

where I is the 512 *×* 512-sized input image and $\hat{I}$ is the reconstructed image. The loss function is calculated using a summation of the output of the logistic units and the pixel intensity and their quadrate differences. Through this process, capsules optimally learn the parameters’ properties for reconstruction, which generalize the ability of the model to learn properties’ parameters with an accuracy almost to the pixel. If the model learns better reconstruction, the output will be with high predictability. Then, the reconstruction loss is input to the next $L_{M}$ margin loss function.

(14) LM=SkΣkmax(0,m+−||υk||)2+λkΣ(1−Sk)max(0,||υk||−m−)2

Here, *S_k_ =* 1 if a pattern of class *k* is present. From here, the momentum in Equation (14), m*^+^ =* 0.9, and the amount of motion is selected *m^−^ =* 0.1. The proposed pass-through architecture is estimated, and the total loss function $L_{T}$, based on weight, which is the sum of all losses of the *k* total classes, is estimated as follows:

(15) $$L_{T}=L_{M}+\xi I_{s}L_{R}$$

Here, *ξ =* 0.0005 is the regularization factor for each channel pixel value, which ensures that restoration loss does not prevail over the $L_{M}$ during training. 

In addition*, I_s_ = h* *× w* *× Ch*—indicates the number of inputs based on height, width, and number of input channels.

## 4. Dataset

**Figure 4:**
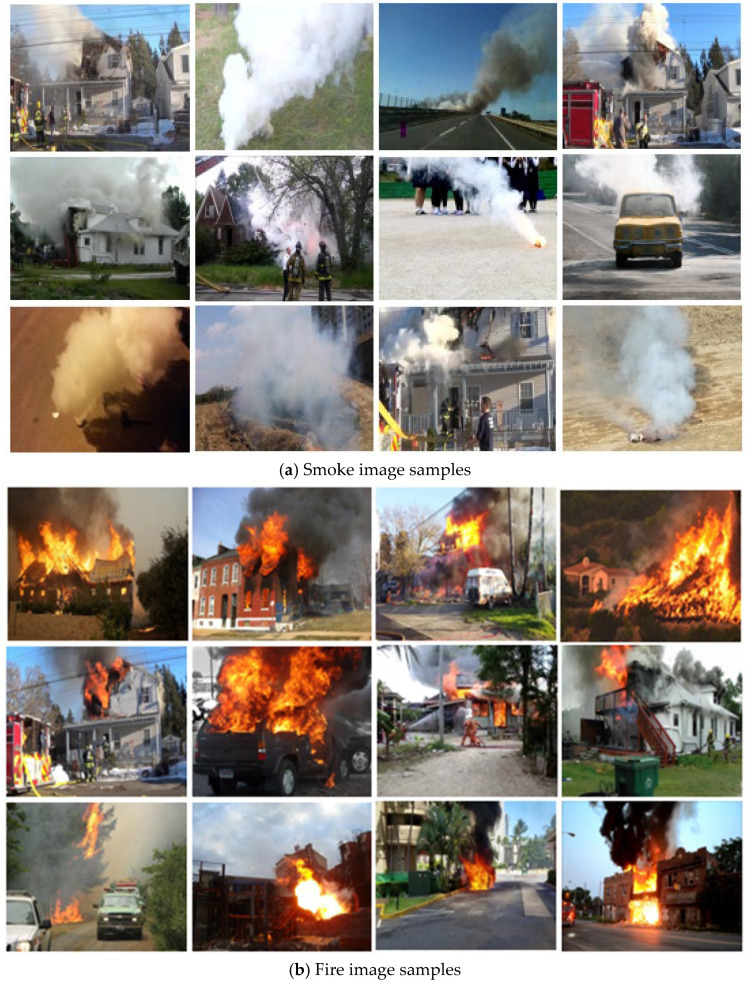

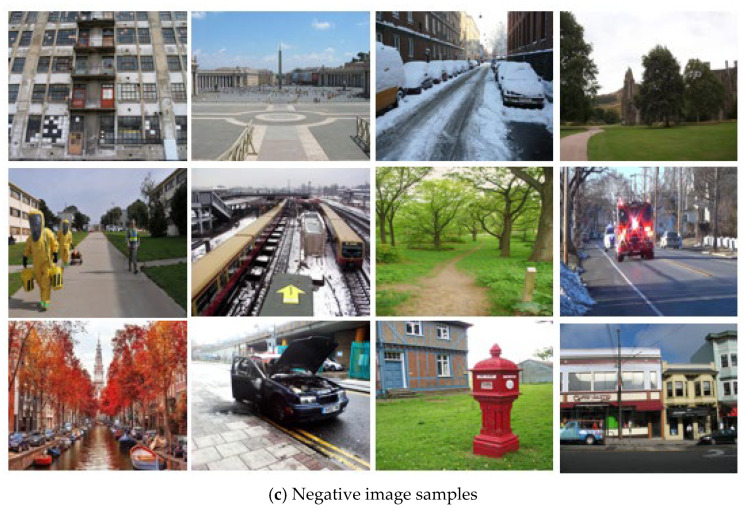
Example images from three classes from datasets for training capsule network.

High-quality fire and smoke datasets are extremely rare and, when open for public use, they are generally of low quality for evaluating and analyzing the proposed methods. Therefore, we mainly collected data from various internet resources to test it on our model. The primary size of our data was from pixels from 512 × 512 images. The datasets contained three main parts: smoke, fire, and negative images. For training and testing, each section contained 4000 images. Table 1 lists the information on a number of all images, as shown in Figure 4, below. We trained and tested the proposed model using our datasets collected from different sources. In total was used 12.000 images. Many of them were opened by the owners for use, and some of them that needed some copyright issues were asked permission for using in our research.

## 5. Experiments and Results

In this section, we describe the experimental setup and generated results individually. First, we prepared our implementation details for training and obtained the results. We then comprehensively discussed our results.

Implementation Details 

The training setup was designed based on the PyTorch framework [34] and trained through the following configuration: Stochastic Gradient Descent (SGD) [35] was used for the backpropagation optimization applied, binary cross-entropy loss function, learning rate (LR) of 0.0005, and 100 epochs. The CPU model of the test equipment was an Intel^®^ Core™ i7-9750H CPU@2.60 GHz, and the software environment was CUDA 10.1, Python 3.7, as portrayed in Table 2.

In addition to these infrastructure changes, we had to perform many tests on various parameters of dozens of tests to train the entire model using different hyper parameters. To accelerate and parallelize the training process, we implemented our CUDA models so that we could use the GPU to reduce the training time. All three authors had access to GPU-related labs where they conducted their thesis. All GPUs were GeForce GTX 1660Ti 16 GB. We spent at least 30 of the superior CapsNet for our results in this article, each of which took about 4 h, so the bottom line of our GPU-hours was 120. We found that changing the standard hyper parameters, describing SGD depending on batch size, learning rate, speed, and momentum, did not significantly affect the final performance. This is because our CapsNets had already come together, so these parameters could only reduce the time spent learning. The hyperparameter that interests us most is the number of iterations of routing from the dynamic routing algorithm. This parameter is unique to CapsNets and has important consequences for their performance and execution. Each time the output is run, multiple routing operations are performed to determine three routing operations. We conducted a number of experiments where we set the number of routing operations per one of 1, 2, 3, 4, and 5. Although 3 iterations gave good test accuracy, in general, 2 performed about some cases, even better. This is interesting for two reasons. First, it contradicts the original article. It may be that 3 iterations converged to become more stable than 2, but we translated our experiments several times and obtained similar graphs each time. Second, it may be indicative that the proposed dynamic routing algorithm was too sharp. The process of determining which primary capsules are inserted into which Digit capsules is complex, and it would be incredible if the algorithm ever needed only two iterations to do this well.

In this section, the classification results of the offered and other classical methods are evaluated. All methods under review have been assessed based on their accuracy (*A*), specificity (*SP*), and sensitivity (*SE*). “*A*” reflects a classifier′s overall effect of prediction. The two variables *SP* and *SE*, respectively, represent the positive and negative predictive power. The following Equation (16) are used to assess the performance of the models under evaluation.

(16) $$A=\frac{TP+TN}{TP+FP+TN+FN}\;\;SE=\frac{TP}{\left(TP+FN\right)}\;\;\;SP=\frac{TN}{FP+TN}$$

where *TP* denotes true positive/blocking, *TN* denotes true negative/nonblocking, *FP* denotes false positive/blocking, and *FN* denotes false-negative/nonblocking.

The power of classification of the models was measured based on the receive operating characteristic curve (AUC) area, which is considered a significant metric to demonstrate the effectiveness of a classification by means of changing the threshold of discrimination. A Matthew Correlation Coefficient (MCC) is another indicator of binary (two-grade) classifications quality. The *MCC* is used to consider the relationships of balancing the four confusion matrix categories, i.e., *TP, TN, FP*, and *FN*, and objectively reflects the models′ predicting power without being influenced by the disproportionate ratio of positives and negatives contained in the dataset. Equation (17) is used to calculate the MCC:

(17) $$MCC=\frac{\left(TP\times TN\right)-\left(FP\times FN\right)}{\sqrt{\left(FP+TN\right)\left(FP+TP\right)\left(FN+TN\right)\left(FN+TP\right)}}$$

The name of the dataset for training was adopted for training all models, and to monitor the training processes, the fivefold cross-validation method was used. The training set was randomly divided into five subsets for fivefold cross-validation. Four of the five subsets were used as the training data. The validation data for testing the model used the remaining subset. The process of cross-validating was repeated six times, and each of the five subsets was used once as the validation data. The average result of the six runs was calculated to obtain a single estimation. 

The results of the fivefold cross-validating process for the training set are shown in Table 3. Based on the given results, the best performance pertains to the CapsNet variable. The overall accuracy prediction *(A)* for the CapsNet model reached 99.4%. It is important to stress that the MCC values of CapsNet accounted for 0.884, respectively, which also constituted the highest of all MCC values (Table 3); in addition, a higher MCC value frequently indicates greater predictive power of the model.

For the purpose of comparing the performance of the given models with other approaches, widely used machine learning techniques were applied to develop predictive models using the same training set of fire data. To this end, such machine learning methods, including a logistic regression (LR), deep belief network (DBN), light gradient boosting machine (LightGBM), multilayer perceptron (MLP), k-nearest neighbors (k-NN), support vector machine (SVM), and convolutional neural network (CNN), were used. The hyper parameters for these methods were optimized using a five-fold cross-validation process beforehand, and the optimal hyper parameters are given in Table 4. Table 4 also lists the results of predictions for the fire dataset name, test set, as well as an external validation set. 

Table 4 demonstrates an obviously higher level of prediction accuracy of the capsule network model with respect to the seven models, mentioned above. Table 4 provides a summary of the results of a capsule network prediction and other popular CNN-based classification models. Table 4 shows that the model demonstrates an outstanding predictive capacity for the fire dataset name, test set, as well as for the external validation set. The accuracy of prediction of the capsule network model was 99.4%, whereas the prediction accuracy of the CNN classification models was at approximately 90.8%.

**Figure 5:**
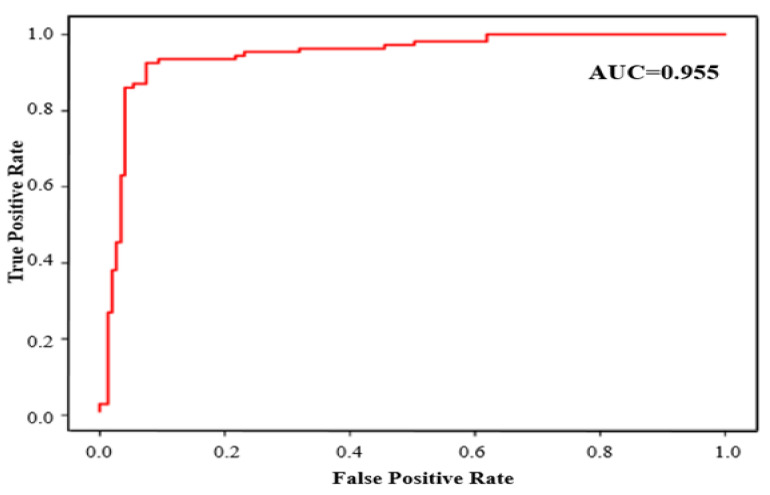
Result of AUC scores of capsule network.

As demonstrated in Figure 5, the AUC values for both the external validation and test sets were at 0.955, respectively. Taken altogether, this unequivocally indicates that the established capsule network can both provide a correct classification for the training set compounds, as well as demonstrate excellent predictability for external agents that are not included in the training set.

**Figure 6:**
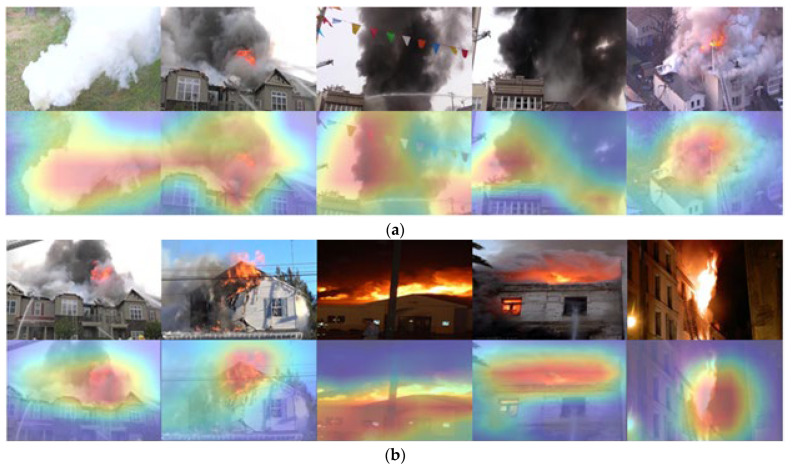

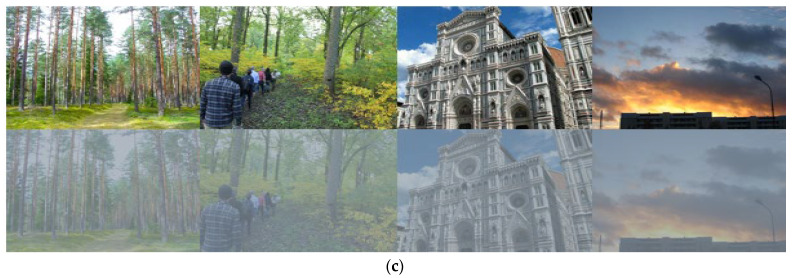
Results of attention feature map using CapsNet for fire classification: (**a**) smoke classification results, (**b**) fire classification results, and (**c**) negative image results.

Figure 6 shows the images of fire and smoke classifications. The method under review showed an outstanding capacity to derive important features from the images. At instances where the images were difficult to differentiate, the features studied by the capsule network were observed at the terminal layers when utilizing the activation map approach. With the recommended method, higher-end results were achieved for the images that looked similar. The proposed capsule network model was proven to be 99.4% greater than other traditional and popular deep models for the images of fire and smoke classification.

In our work, we were able to obtain 30 FPS with our model on the processor. The latest versions of Tensorflow and PyTorch are optimized for performing certain operations on multiple kernels and can be controlled by parameters. Thus, the absence of a GPU does not mean that the process is impossible or time-costly. For example, this may be the case if you are dealing with cloud computing with limited resources.

We also used certain methods to speed up the GPU output even more. In such cases, if the GPU supports fp16, it will simply apply mixed-precision, which is part of the latest versions of PyTorch and TensorFlow. This allows for using the accuracy of fp16 for some layers and fp32 for others, maintaining the numerical stability of the network and thus maintaining accuracy. The alternative and even more effective way to accelerate the model is TensorRT conversion. This is a more complex method, but it can provide a 5x faster output. There are also other obvious optimization options, such as resizing and output. The flexibility of the system was important in this case because we wanted to process not only video files but also different video-recording formats. It showed a good FPS in the range of 30−60 depending on the configuration used.

We previously claimed that the use of the local window size in the capsule layers helps efficiently solve our classification task. However, we do not know exactly how large the capsule should be. We proved that the large size that we chose is the best option for solving the current challenge. The results are shown in Figure 6.

## 6. Conclusions

In this paper, our proposed model is described for the visible detection of smoke and fire classification using an attention feature map based on capsule networks. Our model has a robust design that allows firefighters to categorize outdoor CCTV images in real-time. Using this proposed approach, we applied the following main components: an attentional function map, large-sized input images, high features, and a local window. We showed experimentally that the current problem can be efficiently solved using these contributions. The proposed model is simple to design and can be trained fast. The proposed solution achieved promising results for accuracy in comparison to the state-of-the-art. In terms of false positives, it reduced the background errors in nonfire and smoke videos. The proposed method fulfilled the high accuracy, even with videos consisting of challenging features such as the sun and clouds. The proposed work solution is suitable for its low-resolution and real-time performance when compared to the other methods. Our results showed that the model can detect smoke and fire in a short period of time as an early alerting alarm for the occasion of fire and smoke accidents. To evaluate the performance of our model and to compare it with other approaches, we experimented with a custom dataset, which contained highly variable images, occlusions, different viewpoints, and lighting and weather conditions such as rainy, cloudy, and sunny days. We conducted experiments comparing the performance and generalizability of our approach with other current methods. These experimental results confirmed that our proposed capsule network method is the best among different well-known architectures. Our model provides higher accuracy for completely new images than previous approaches. The proposed method is effective, allowing researchers to detect fires at an early stage, determine the location of a fire, and save the lives and property of people.

In a future study, we will explore expansion models more efficiently and in detail with thermal cameras or drone feature representations, and we look forward to improving the model by applying a 3D convolutional neural network, where a 3D network obtains features from both extensional and temporal dimensions based on 3D convolutions.

## Figures and Tables

**Table 1:** Dataset information.

Name of Class Images	Smoke	Fire	Negative	Amount
Dataset	4000	4000	4000	12,000

**Table 2:** Performance hardware and software of computer.

Technology	Description
Programming language	Python 3.7
OS	Windows
Deep Learning library	PyTorch
CPU	Intel(R) core™ i-7 9750H
GPU	GeForce GTX 1660 Ti
RAM	16.00 GB RAM
Cuda	10.1

**Table 3:** Comparing results with a different architecture.

Capsule Network Architecture	SE	SP	MCC	A (%)
Original CapsNet	80.4%	86.7%	0.673	84.1%
FC+FC	82.6%	86.7%	0.694	85.0%
Conv+FC	82.6%	84.6%	0.687	84.6%
Conv+FC+FC	84.5%	85.3%	0.693	84.9%
Conv+Conv+FC+FC (our model)	99.0%	99.7%	0.884	99.4%
Conv+Conv+Conv+FC+FC	81.9%	86.9%	0.685	84.9%

**Table 4:** Comparison of accuracies of the same training dataset of smoke and fire classification for different methods.

Model	SE	SP	MCC	A (%)	AUC
**Test Set**					
Our Model	91.8%	92.9%	0.850	92.4%	0.955
CNN [36]	87.0%	85.0%	0.715	85.9%	0.933
MLP [37]	82.4%	86.4%	0.687	84.7%	0.920
DBN [38]	72.2%	80.8%	0.533	80.8%	0.903
SVM [39]	90.7%	84.4%	0.743	87.1%	0.933
k-NN [40]	69.4%	96.6%	0.703	85.1%	0.928
Logistic regression [41]	88.8%	83.7%	0.710	85.5%	0.858
LightGBM [42]	79.6%	82.3%	0.617	81.2%	0.810
**Validation Set**					
Our Model	88.9%	71.4%	0.554	76.7%	0.806
CNN	94.4%	52.4%	0.441	65.0%	0.725
MLP	88.9%	57.1%	0.426	66.7%	0.707
DBN	88.9%	52.4%	0.386	63.3%	0.683
SVM	88.9%	52.4%	0.386	63.3%	0.660
k-NN	77.8%	52.4%	0.279	60.0%	0.624
Logistic regression	83.3%	52.4%	0.332	61.7%	0.623
LightGBM	61.1%	59.5%	0.190	60.0%	0.609

The MCC values and overall accuracy of prediction (*A*) were 92.4%.

## Data Availability

Not applicable.
